# Low-fecundity *dhc-1; mel-28 C. elegans* mutants do not have gonad mitosis defects

**DOI:** 10.17912/micropub.biology.001445

**Published:** 2025-01-21

**Authors:** Julia Stobierska, Anita G. Fernandez

**Affiliations:** 1 Biology, Fairfield University, Fairfield, Connecticut, United States

## Abstract

In *C. elegans , dhc-1 ( or283 ); mel-28 ( t1684 )* double mutants have a severely reduced brood size compared with each single mutant and compared to the wild type. To determine if this synthetic low-fecundity phenotype is due to reduced potential to produce gametes, we studied gonad length and distal gonad mitotic activity in *dhc-1 ( or283 )* mutants, *mel-28 ( t1684 )* mutants, wild-type animals, and *dhc-1 ( or283 ); mel-28 ( t1684 )* double mutants. Gonad length in *dhc-1 ; mel-28* double mutants was the same as the wild type. Using an antibody against phosphorylated histone H3 (PH3), we tracked mitotic activity in mutant and wild-type gonads. We found no significant difference in mitotic activity between the double mutant and the wild-type. These observations suggest that the reduced brood size in *dhc-1 ; mel-28* double mutants is not caused by a mitotically-inactive gonad and instead has a different and yet-to-be-determined basis.

**Figure 1. dhc-1; mel-28 double mutant gonads resemble the wild type with regard to length and mitotic activity:**
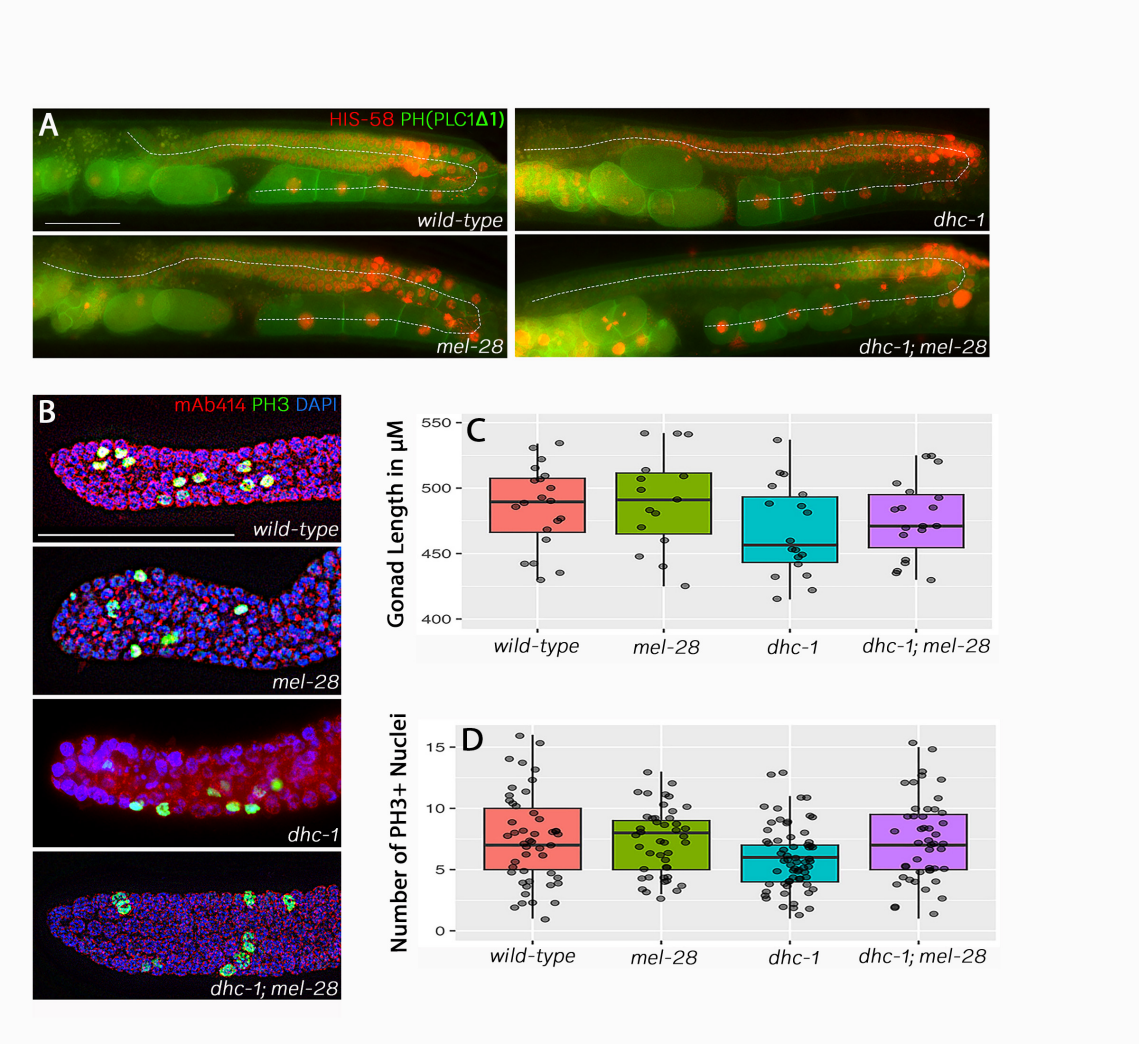
(A) Gonads from live wild-type animals, *mel-28* mutants, *dhc-1* mutants, and *dhc-1 ; mel-28* double mutants. Red marks the chromatin and green marks the cell membranes. Z-stacks were captured and then collapsed using the Keyence microscope Analyzer software. The dotted lines indicate how we measured the length of the gonads. Scale bar = 50 μM (B) Fixed distal gonads from wild-type animals, *mel-28* mutants, *dhc-1* mutants, and *dhc-1 ; mel-28* double mutants. The blue represents DAPI staining, the red indicates nuclear pores, and the green indicates mitotic nuclei. Z stacks were captured and collapsed, and images were processed using haze-reduction software from the Keyence Analyzer package. Scale bar = 50 μM. (C) Gonad arm length measurements. At least 20 gonads of each strain were measured. Pairwise t tests between strains yielded p values > 0.05, except for the comparison between *mel-28* mutant gonads and *dhc-1* mutant gonads, which returned a p value of 0.014. (D) PH3-positive nuclei counts. At least 60 gonads of each genotype were analyzed for mitotic activity. Pairwise t tests between the strains yielded p values > 0.05, except for the comparison between *dhc-1* mutants and the wild-type which showed a p value of 0.048.

## Description

The simultaneous disruption of dynein components and the nucleoporin MEL-28 causes fertility defects in *C. elegans* (Fernandez, et al., 2014, Gandhi et al., 2021) . When both genes are disrupted at 26 °C, the average hermaphrodite brood size is about thirty embryos whereas each single mutant usually produces close to one hundred embryos (Fernandez et al, 2014) . This suggests that dynein and MEL-28 act in parallel to promote fertility. Dynein is a multi-component motor required for minus-end-directed intracellular trafficking in animals (Pfister et al., 2006, Canty et al., 2021) MEL-28 is a conserved nucleoporin associated with the Y subcomplex of the nuclear pore, and has roles in both the post-mitotic rebuilding of the nuclear pore and in chromatin state (Galy et al. 2006, Fernandez and Piano 2006, Doucet et al., 2011) .

The *C. elegans* gonad consists a two-armed tube. The distal tip cell caps each gonad arm and acts via Notch signaling to maintain a mitotically dividing stem cell niche (Austin and Kimble, 1987) As nuclei move toward the proximal end of the gonad, they commit to gamete fate by discontinuing mitosis and entering prophase I of meiosis. Disruption of gonad mitotic activity, for example via reduction of Notch signaling ( Austin and Kimble, 1987 ; Yochem and Greenwald, 1989 ) reduces cell division in the distal gonad and limits the number of gametes produced. We decided to test whether the reduced fecundity we observe in *dhc-1 ; mel-28* double mutants was coincident with reduced mitotic potential in the germ line.

To better visualize the gonad in live animals, we first generated mutant strains expressing mCherry:: HIS-58 and GFP::PH(PLC1delta1), each from the germ-line-specific *pie-1* promoter (Essex et al., 2009) . We then measured gonad arm length in wild-type, *dhc-1* single mutant, *mel-28* single mutant, and *dhc-1 ; mel-28* double mutant hermaphrodite adults ( Figure 1A and 1C). The gonad arm length was not significantly different between the wild-type and any of the mutant strains we examined. We did observe that *mel-28* single mutant gonad arms were slightly longer than *dhc-1* single mutant gonad arms (p= 0.014). Next we studied mitotic events in the stem cell niche of each mutant. Phosphorylated histone H3 (PH3) decorates the chromatin of M-phase nuclei (Hendzel et al, 1997) . To determine if there were differences in mitotic activity amongst the strains, we counted the number of PH3-positive nuclei in the distal gonad of each mutant strain ( Figure 1B ). We found that *dhc-1 ; mel-28* double mutants did not have a reduced number of mitotic nuclei compared to the wild-type ( Figure 1D ), suggesting that mitotic potential is unaffected in these mutants. We did observe a slight reduction in the average number of M-phase nuclei in *dhc-1* single mutants compared to the wild type (p= 0.048). Based on these results, we can definitively rule out the idea that insufficient production of germ cells causes the low fecundity we observe in *dhc-1 ; mel-28* double mutants.

## Methods

Gonad measurements:

The *dhc-1 ( or283 )* mutant allele we used is temperature sensitive, which allows animals to thrive at 16 °C and causes embryonic lethality at 26 °C (Hamill et al., 2002). All animals were maintained at 16 °C. L4 hermaphrodites were placed to 26 °C for 24 hours before imaging. Adults were put in 2 mM levamisole and then on 2% agar pads on a glass slide. These were imaged at 40X using a Keyence BZ-X800 microscope. We used the Keyence Analyzer software (version 1.1.0.23) to measure each gonad by drawing a line from the -1 oocyte to the distal tip of the gonad.

Immunolocalizations:

L4 hermaphrodites were placed to 26 °C for 24 hours before imaging. Adults were dissected on a polylysine-coated slide in egg buffer (25 mM HEPES pH 7.4, 118 mM NaCl, 48 mM KCl, 2 mM CaCl2, 2 mM MgCl2) with 2 mM levamisole. A coverslip was placed over the dissected animals and then the slide was immediately immersed in liquid nitrogen. After abruptly removing the coverslip from the frozen slide, slides were placed in methanol at -20 °C for ten minutes, followed by acetone at -20 °C for five minutes, followed by an ice-cold acetone hydration series. After a PBS wash, slides were incubated with primary antibodies (1:200 Rabbit anti-phosphorylated histone H3 and 1:400 mAb414 to identify the nuclear peripheries) in a humid chamber at room temperature overnight. Slides were washed three times in PBST and incubated with secondary antibodies (1:400) in PBS in a humid chamber for one hour. Slides were washed three times in PBST and mounted using Vectashield Plus Antifade Mounting medium with DAPI and sealed with nail polish. Imaging was done at 60X using a Keyence BZ-X800 microscope. We captured Z stacks in each channel and manually counted the number of PH3-positive nuclei in each stack. All images were processed using Adobe Photoshop (24.12.1 release).

## Reagents

*C. elegans* strains

**Table:** 

**Strain name**	**Strain genotype**	**availability**
OD95	*unc-119 ( ed3 ) III; ltIs37 [ pie-1 p::mCherry:: his-58 + unc-119 (+)] IV. ltIs38 [ pie-1 p::GFP::PH(PLC1delta1) + unc-119 (+)]. *	CGC
AGF110	*dhc-1 ( or283 ) I; mel-28 ( t1684 )/ qC1 III; ltIs37 [(pAA64) pie-1 p::mCherry:: his-58 + unc-119 (+)] IV. ltIs38 [ pie-1 p::GFP::PH(PLC1delta1) + unc-119 (+)]. *	Upon request
AGF135	*mel-28 ( t1684 )/ qC1 III; ltIs37 [(pAA64) pie-1 p::mCherry:: his-58 + unc-119 (+)] IV. ltIs38 [ pie-1 p::GFP::PH(PLC1delta1) + unc-119 (+)]. *	Upon request
AGF140	*dhc-1 ( or283 ) I; mel-28 ( t1684 )/ qC1 III; ltIs37 [(pAA64) pie-1 p::mCherry:: his-58 + unc-119 (+)] IV. ltIs38 [ pie-1 p::GFP::PH(PLC1delta1) + unc-119 (+)]. *	Upon request
N2	wild type	CGC
AGF001	*mel-28 ( t1684 )/ qC1 III *	Upon request
AGF035	*dhc-1 ( or283 ) I; mel-28 ( t1684 )/ qC1 III *	Upon request
EU1385	*dhc-1 ( or283 ) I *	CGC

Antibodies

**Table:** 

**Antibody**	**Source**
mAb414 (to detect FG-repeat nuclear pores)	AbCam ab24609
Anti-Phosphorylated Histone H3 (S10)	AbCam ab5176
Goat anti-mouse TRITC	Jackson 115-025-003
Goat anti-rabbit FITC	Jackson 111-095-003
